# Effects of improved water, sanitation, and hygiene and improved complementary feeding on environmental enteric dysfunction in children in rural Zimbabwe: A cluster-randomized controlled trial

**DOI:** 10.1371/journal.pntd.0007963

**Published:** 2020-02-14

**Authors:** Ethan K. Gough, Lawrence H. Moulton, Kuda Mutasa, Robert Ntozini, Rebecca J. Stoltzfus, Florence D. Majo, Laura E. Smith, Gordana Panic, Natasa Giallourou, Mark Jamell, Peter Kosek, Jonathan R. Swann, Jean H. Humphrey, Andrew J. Prendergast, for the Sanitation Hygiene Infant Nutrition Efficacy (SHINE) Trial Team

**Affiliations:** 1 Department of International Health, Johns Hopkins Bloomberg School of Public Health, Baltimore, MD, United States of America; 2 Zvitambo Institute for Maternal and Child Health Research, Harare, Zimbabwe; 3 Division of Nutritional Sciences, Cornell University, Ithaca, NY, United States of America; 4 Department of Epidemiology and Environmental Health, School of Public Health and Health Professions, University at Buffalo, Buffalo, NY, United States of America; 5 Department of Metabolism, Digestion and Reproduction, Division of Digestive Diseases, Faculty of Medicine, Imperial College London, London, United Kingdom; 6 Pain Care Specialists of Oregon, Eugene, OR, United States of America; 7 Department of Neuroscience, Karolinska Institute, Solna, Sweden; 8 Blizard Institute, Queen Mary University of London, London, United Kingdom; Massachusetts General Hospital, UNITED STATES

## Abstract

**Background:**

Environmental enteric dysfunction (EED) may be an important modifiable cause of child stunting. We described the evolution of EED biomarkers from birth to 18 months in rural Zimbabwe and tested the independent and combined effects of improved water, sanitation, and hygiene (WASH), and improved infant and young child feeding (IYCF), on EED.

**Methodology and findings:**

The Sanitation Hygiene Infant Nutrition Efficacy (SHINE) trial was a 2x2 factorial cluster-randomised trial of improved IYCF and improved WASH on child stunting and anaemia at 18 months of age. 1169 infants born to HIV-negative mothers provided plasma and faecal specimens at 1, 3, 6, 12, and 18 months of age. We measured EED biomarkers that reflect all domains of the hypothesized pathological pathway. Markers of intestinal permeability and intestinal inflammation declined over time, while markers of microbial translocation and systemic inflammation increased between 1–18 months. Markers of intestinal damage (I-FABP) and repair (REG-1β) mirrored each other, and citrulline (a marker of intestinal epithelial mass) increased from 6 months of age, suggesting dynamic epithelial turnover and regeneration in response to enteric insults. We observed few effects of IYCF and WASH on EED after adjustment for multiple comparisons. The WASH intervention decreased plasma IGF-1 at 3 months (β:0.89, 95%CI:0.81,0.98) and plasma kynurenine at 12 months (β: 0.92, 95%CI:0.87,0.97), and increased plasma IGF-1 at 18 months (β:1.15, 95%CI:1.05,1.25), but these small WASH effects did not translate into improved growth.

**Conclusions:**

Overall, we observed dynamic trends in EED but few effects of IYCF or WASH on biomarkers during the first 18 months after birth, suggesting that these interventions did not impact EED. Transformative WASH interventions are required to prevent or ameliorate EED in low-income settings.

## Author summary

Child stunting remains a global health challenge rooted in an intergenerational cycle of poor health, reduced neurodevelopment and poverty. Environmental enteric dysfunction (EED) is an acquired condition of the small intestine likely resulting from frequent faecal-oral microbial exposure, which is hypothesized to underlie stunting. We found dynamic changes in EED biomarkers between 1 and 18 months of age in a cohort of rural Zimbabwean infants, suggesting a complex developmental period of intestinal maturation, adaptation and response to environmental insults. Randomized improved infant and young child feeding, and improved water, sanitation and hygiene (WASH) interventions had no meaningful impact on EED. Greater investment in transformative WASH is needed to prevent EED in low-income countries.

## Introduction

Child stunting, defined as an attained length or height >2 standard deviations below the age- and sex-matched standard population median [1], is a persistent global health challenge. Stunting is associated with reductions in child survival, early childhood development, educational attainment and adult economic productivity [2–4]. An estimated one-fifth of children (149 million) are stunted before the age of 5 years in low- and middle-income countries (LMICs) [5]. Interventions to reduce stunting have largely focused on improved complementary feeding and prevention of diarrhoea. However, the impact of these interventions on restoring linear growth deficits is modest [6–8]. Evidence to support other nutrition-sensitive interventions, which address the underlying determinants of stunting during the first 1000 days of life, is currently limited [9]. The need to identify modifiable causal mechanisms underlying stunting is a paramount global health need.

Three decades ago, tropical enteropathy was proposed as an important factor in undernutrition [10,11]. This small intestinal pathology, now called environmental enteric dysfunction (EED), has recently gained considerable interest as a plausible cause of stunting [12,13]. EED is a subclinical disorder characterized by reduced villus height, increased crypt depth and lymphocytic infiltration [12], resulting in impaired absorption and gut barrier function. Nutrient deficiencies, fungal toxin exposure and chronic exposure to enteric pathogens are hypothesized causes of EED [14–16].

There is no agreed case definition for EED. Differential urinary excretion of two sugars, typically lactulose and mannitol, has most often been used to characterize abnormalities in gut permeability and absorption, but this test has substantial limitations [17,18] and only captures part of the pathology underlying EED [15]. To date, studies have failed to demonstrate reproducible changes in these markers following interventions. Nutritional supplementation with vitamin A [19], zinc [20,21], multiple micronutrients [22], alanlyl-glutamine [23–25] or dietary supplementation [26,27] have produced transient or inconsistent improvements. Interventions aimed at ameliorating intestinal inflammation, permeability or microbiome disruption have also been unsuccessful, including probiotics [28], antibiotics [29], long-chain polyunsaturated fatty acids [30], lactoferrin with lysozyme [31], and albendazole plus zinc [32]. Several other biomarkers have been adopted to reflect a wider range of functional and structural characteristics of EED [15,33]. A number of these have shown modest negative relationships with linear growth, although results vary by setting and age group [32,34–40]. Since EED is acquired early in life among children living in impoverished unsanitary conditions [15,41,42], it is plausible that water, sanitation and hygiene (WASH) interventions, designed to block faecal-oral transmission of enteropathogens, may prevent EED. The WASH Benefits trial in Bangladesh, testing the impact of a water, sanitation and hygiene intervention with or without a nutritional supplement, showed that the interventions improved some markers of gut inflammation and permeability in infants at three months of age, but by 28 months of age several biomarkers were higher among infants in the intervention arms compared to control [43]. In addition, only the MAL-ED study has measured the range and dynamics of EED biomarkers during the first two years of life, and only for a limited set of markers [44].

The Sanitation Hygiene Infant Nutrition Efficacy (SHINE) trial [45] was a cluster-randomized trial testing the impact of improved household water quality, sanitation, and hygiene (WASH) and improved infant and young child feeding (IYCF) on linear growth between birth and 18 months. We hypothesized that the effect of the WASH intervention would be mediated in part through reduced diarrhoea but primarily through reduced EED. We have previously reported the trial primary outcomes: the IYCF intervention modestly increased length-for-age Z-score (LAZ) by 0.16 and reduced stunting by 20% at 18 months, but the WASH intervention had no impact on linear growth or diarrhoea [46]. The WASH Benefits trials in Bangladesh and Kenya also found no impact of a WASH intervention on stunting and WASH Benefits Kenya found no impact on diarrhoea [47,48]. To investigate the impact of the WASH and IYCF interventions on EED, and to describe the evolution of EED during infancy, we measured biomarkers of EED in a subgroup of infants enrolled in SHINE at 1, 3, 6, 12, and 18 months of age.

## Methods

A detailed description of the SHINE trial design and methods has been published [45], and the protocol, behaviour change modules, and statistical analysis plan are available at https://osf.io/w93hy. SHINE was a 2x2 factorial cluster-randomised trial assessing the individual and combined effects of improved IYCF and improved WASH on child stunting and anaemia at 18 months of age (ClinicalTrials.gov NCT01824940). A total of 211 clusters, defined as the catchment area of 1–4 Village Health Workers (VHWs) employed by the Ministry of Health and Child Care (MoHCC), were allocated to one of four intervention groups using highly constrained randomization: standard-of-care (SOC); IYCF; WASH; or IYCF+WASH. Between 22 November 2012 and 27 March 2015, pregnant women living in these clusters were enrolled following written informed consent. VHWs delivered treatment group-specific behavior change interventions and commodities during 15 home visits, between enrolment and 12 months postnatal. Between 13–17 months, VHWs made monthly visits to provide routine care, deliver intervention commodities in active groups, and encourage participants to practice behaviors relevant to their study arm. At 18 months a review module, which reiterated key messages, was implemented in all arms. Commodities and behavior change communication messages delivered in the four treatment groups were:

*SOC*: promotion of exclusive breastfeeding to 6 months [49], and uptake of maternal-child health services.

*WASH*: SOC interventions plus a ventilated improved pit (VIP) latrine, two handwashing stations, plastic mat and play yard (North States, Minneapolis, MN); monthly delivery of soap and chlorine solution (WaterGuard, Nelspot, Zimbabwe) with promotion of safe disposal of feces, handwashing with soap, protection of infants from geophagia, chlorination of drinking water, and hygienic preparation of complementary food.

*IYCF*: SOC interventions plus 20g small-quantity lipid-based nutrient supplement (SQ-LNS) to be fed to the infant daily from 6–18 months; education and counseling to feed the infant nutrient-dense locally available food, feeding during illness, and dietary diversity.

*WASH+IYCF*: All SOC, WASH, and IYCF interventions.

A latrine was constructed in SOC and IYCF arms after completion of the trial. Due to the nature of the interventions, masking was not possible.

### Data collection

Research nurses made two home visits during pregnancy and five postnatal visits at infant ages 1, 3, 6, 12, and 18 months. At baseline, maternal education and age, household wealth, access to water and sanitation, and food insecurity were assessed; mothers were tested for HIV via rapid test algorithm and HIV-positive women were urged to seek immediate care for prevention of mother-to-child transmission. Infant birth date, weight, and delivery details were transcribed from health facility records; the trial provided Tanita BD-590 infant scales to all health institutions in the study area and trained facility staff. Gestational age at delivery was calculated from the date of the mother’s last menstrual period.

Given the household-based trial interventions, visits were not conducted if the mother had moved from the household where she consented, except for the 18-month visit (trial endpoint) when follow-up was conducted anywhere within Zimbabwe. Indicators of uptake of the interventions were collected at all visits and reported here for the 12-month postpartum visit.

#### Environmental Enteric Dysfunction (EED) substudy

Part-way through the trial (from mid-2014 onwards) mother-infant pairs were invited to join a substudy to investigate biomarkers of EED. Women were informed about the EED substudy at their 32-week gestation visit and those with live births were enrolled at the 1 month postnatal visit, or as soon as possible thereafter. We pre-specified that primary trial inferences would be based on findings among infants born to mothers who were HIV-negative during pregnancy, because of the likely impact of HIV exposure and infant cotrimoxazole prophylaxis on underlying causal pathways [33]. Results from HIV-unexposed children are reported in this paper; EED results among HIV-exposed infants will be reported separately.

From children in the EED substudy, additional specimens were collected at each postnatal visit, including stool (passed on the morning of the research visit and collected by the mother into a plain container) and blood, collected by venepuncture into an EDTA tube. In the field laboratory, EDTA tubes were centrifuged to collect plasma, which was stored at -80°C. Stool specimens were transported in a cold box to the field laboratory, aliquoted into plain tubes and stored at -80°C. Samples were subsequently transferred to the Zvitambo Laboratory in Harare for long-term storage at -80°C until analysis.

#### Biomarkers of EED

We selected a range of biomarkers that would characterize the domains of EED. These were previously described, together with the rationale for each [33], with the following modifications since publication of that methods paper [50]. First, plasma citrulline was added as a biomarker of small intestinal damage, since lower circulating concentrations reflect reduced enterocyte mass [51]. Second, EndoCAb was dropped as a measure of microbial translocation due to technical problems with the commercial assay [50]. Third, kynurenine:tryptophan ratio was added as an emerging marker of inflammation in children with EED [37], in place of alpha-1-acid glycoprotein which had a high coefficient of variation in our laboratory. Lactulose-mannitol testing was undertaken at all time-points except 1 month of age, because of concerns about interrupting early exclusive breastfeeding. The test has previously been described in detail elsewhere [33]. Briefly, after a 30-minute fast infants ingested 2mL/kg body weight (maximum 20mL) of a sterile solution containing 250 mg/mL lactulose and 50 mg/mL mannitol. A urine bag was placed, and all urine passed over a 2-hour period was collected, preserved with chlorhexidine and transported in a cool box to the field laboratory, where it was measured, aliquoted into plain tubes and stored at -80C. The final list of EED biomarkers and their respective domains are summarized in Table 1.

**Table 1:** Domains of environmental enteric dysfunction and corresponding biomarkers.

Domain	Biomarker
Intestinal Inflammation	Fecal neopterin, fecal myeloperoxidase
Small Intestinal Damage and Repair	Fecal regenerating gene 1β, plasma intestinal fatty acid binding protein, plasma citrulline
Intestinal Permeability	Fecal alpha-1 antitrypsin, urinary lactulose:mannitol ratio
Microbial Translocation	Plasma soluble CD14
Systemic Inflammation	Plasma C-reactive protein, plasma kynurenine:tryptophan ratio
Growth Axis	Plasma insulin-like growth factor 1

All assays were undertaken by laboratory scientists masked to the trial intervention arm. Plasma samples were tested at the Zvitambo laboratory by Enzyme Linked Immunosorbent Assay (ELISA) according to manufacturers’ instructions for CRP (limit of detection (LOD) 0.01ng/mL), soluble CD14 (LOD 125pg/mL), IGF-1 (LOD 0.026ng/mL) (all from R&D Systems, Minneapolis, MN, USA); and I-FABP (LOD 47pg/mL); Hycult Biotechnology, Uden, The Netherlands. Stool samples were tested at the Zvitambo laboratory by ELISA according to manufacturers’ instructions for neopterin (LOD 0.7nmol/L; GenWay Biotech Inc, San Diego, CA, USA), myeloperoxidase (LOD 1.6ng/mL; Immundianostik, Bensheim, Germany), A1AT (LOD 1.5ng/mL; BioVendor, Brno, Czech Republic), and REG‐1β (LOD 0.625ng/mL; TECHLAB Inc, Blacksburg, VA, USA). Plasma citrulline (LOD 100ng/mL), kynurenine (LOD 40ng/mL), and tryptophan (200ng/mL) were assayed by ultrahigh-performance liquid chromatography tandem mass spectrometry with electrospray ionization (Waters, Wilmslow, U.K.) at Imperial College, London. Urine samples were tested on a Shimadzu Prominence liquid chromatograph with a Restek Ultra Amino 3μm 150/2.1mm column and tandem Sciex QTRAP5500 mass spectrometer with a Turbo V ion source and TurboIonSpray probe at Pain Care Specialists of Oregon, USA. Both analytes were quantified against lab-made calibrators at ng/mL levels of 3250, 2500, 1250, 750, 350, 100, 10, and 1. The calibrator was confirmed each day by lab-made quality control samples at 150 and 750ng/mL. Lactulose for calibrators and control were obtained from Spectrum Chemical (LOD 1ng/mL). Mannitol was obtained from Tokyo Chemical Industry (LOD 1ng/mL). Both internal standards were obtained from Sigma-Aldrich. Citrulline and KTR were not measured at 18 months (no funding for samples). We also calculated the environmental enteropathy score (EE score) proposed by Kosek *et al*. [52], as a composite index reflecting the severity of intestinal enteropathy and treated it as a continuous variable in our analyses.

#### Sample size

The substudy was based on a sample size of 250 children per trial arm with longitudinal assessments of EED, after allowing for missed samples and loss to follow-up. Assuming an average of 2–3 infants per cluster, type I error of 5%, and a coefficient of variation across clusters of 0.25 provides >80% power to detect a difference of at least 0.18 standard deviations between WASH and non-WASH arms, or between IYCF and non-IYCF arms.

### Statistical methods

All analyses were intention-to-treat at the child level. Independent intervention effects of IYCF and WASH, as well as IYCF-by-WASH interaction effects, were evaluated by fitting separate regression models with each biomarker as the dependent variable. Independent variables included IYCF and WASH coded as dummy variables and an IYCF-by-WASH interaction term. If the interaction was not significant (p>0.05) and if the interaction term was <0.25 standard deviations in absolute magnitude, main intervention effects were tested to compare IYCF to non-IYCF and WASH to non-WASH groups in separate models. Except for citrulline and EE score, tobit regression was used to account for unobserved biomarker values below the LOD using the package *AER* [53], and sandwich standard error estimation was used to account for cluster membership using the *sandwich* package [54], both in R version 3.5.3. For citrulline and EE score, no infants were below the LOD, so linear regression models were fitted by generalized estimating equations (GEE), with an exchangeable correlation to account for cluster membership, using the *geepack* package [55]. All biomarker values were natural log-transformed, except for EE score because of zero values. A separate model was fitted for each study visit. For each biomarker, the Holm method was used to account for multiple comparisons across study visits. The robustness of model results to influential observations was assessed by re-fitting each model after 95% winsorization of log-transformed biomarker values or raw EE scores [56]. Regression coefficients are reported as the ratio of biomarker concentrations between intervention and control arms, or as the difference between arms for EE scores. To graphically present the longitudinal biomarker trajectories in each intervention group, we fitted generalized additive models of biomarker concentration against infant age with the use of cubic splines with 3 knots for smoothing.

### Ethics and regulatory oversight

Women gave written informed consent to join the trial and additional consent for their infant to join this substudy. Both the SHINE trial and this substudy were approved by the Medical Research Council of Zimbabwe and the Institutional Review Board of the Johns Hopkins Bloomberg School of Public Health.

## Results

**Fig 1:**
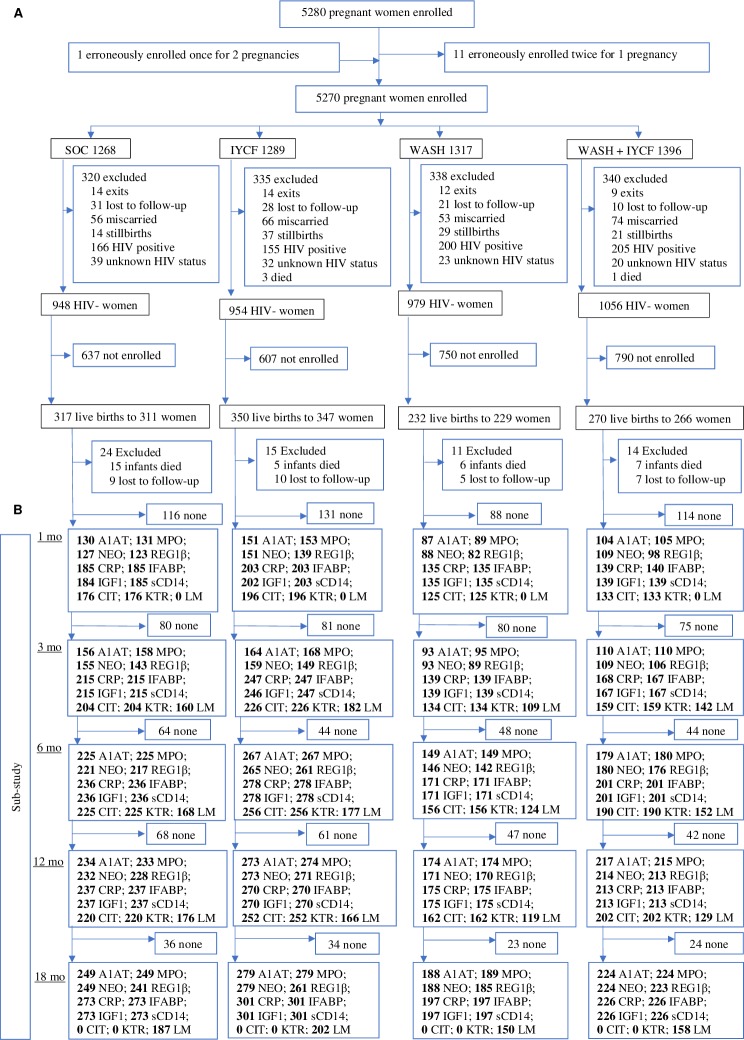
Flow of participants through the SHINE Environmental Enteric Dysfunction (EED) substudy. (A) Enrolment, treatment allocation, sub-study selection and losses to follow-up. (B) Specimen collection. SOC, standard of care; IYCF, infant and young child feeding; WASH, water, sanitation and hygiene. None means that no serum or stool samples were collected at that visit, but infants remained in the substudy.

Of 5280 women enrolled in the SHINE trial, there were 3989 live births to 3937 HIV-negative mothers; of these, 1169 (29%) infants were enrolled in the EED substudy. Of the 1169 enrolled infants, 33 (2.8%) died and 31 (2.7%) were lost to follow-up or exited before the 18-month visit (Fig 1A). A further 68 did not provide a specimen at 18 months, meaning 1037 infants were included in analyses (Fig 1B). Specimen collection at interim visits was lowest at the earliest study visit and was largely similar across intervention arms (Fig 1B).

Baseline characteristics of mother-infant pairs who were enrolled versus not enrolled in the EED substudy are shown in S1 Table. Compared with mothers not enrolled in the substudy, EED mothers had a slightly better food security (assessed by the coping strategy index) and were from households with a more diverse diet. Baseline characteristics by randomized arm for the 1037 infants included in this analysis are shown in Table 2. More than half of all household members practiced open defecation and about one-third of households had an improved latrine at baseline. Approximately two-thirds obtained drinking water from improved sources, and the median return-trip walk from their water source was ten minutes. The frequency of open defecation was higher, while improved latrine ownership, wealth index scores, and employment rate were lower in the SOC arm than in the other arms. Maternal, household and infant baseline characteristics were otherwise largely similar across intervention arms.

**Table 2:** Maternal, household and infant baseline characteristics of HIV-negative mothers and their liveborn infants included in analyses, by intervention arm.

	SOC	IYCF	WASH	IYCF plus WASH
Mothers	311	347	229	266
Infants	279	316	206	245
Mothers completing baseline visit	275	313	203	241
Household Characteristics				
Median Number of Occupants [IQR]	5[3,6]	5[4,6]	5[4,6]	5[4,6]
Wealth Quintile[57]				
1 (lowest)	59 (23.0%)	40 (13.3%)	39 (19.7%)	37 (15.8%)
2	56 (21.8%)	58 (19.3%)	37 (18.7%)	48 (20.5%)
3	51 (19.8%)	63 (20.9%)	43 (21.7%)	50 (21.4%)
4	47 (18.3%)	70 (23.3%)	46 (23.2%)	47 (20.1%)
5 (highest)	44 (17.1%)	70 (23.3%)	33 (16.7%)	52 (22.2%)
Electricity				
Power grid	8/255 (3.1%)	11 (3.7%)	5 (2.5%)	1 (0.4%)
Other power source				
Solar	177/255 (69.4%)	228 (76.0%)	141 (71.2%)	181 (77.0%)
Generator	4/255 (1.6%)	6 (2.0%)	7 (3.5%)	6 (2.6%)
No Electricity	74/255 (29.0%)	66 (22.0%)	50 (25.3%)	48 (20.4%)
Sanitation				
Household members defecate in the open	99/801 (12.4%)	134/884 (15.2%)	94/576 (16.3%)	116/642 (18.1%)
Any latrine at household	82/255 (32.2%)	122 (41.1%)	65 (34.4%)	79 (35.0%)
Improved latrine at household	69/255 (27.1%)	103 (34.7%)	59 (31.4%)	71 (31.4%)
Improved latrine with well-trodden path not shared with other households and not used for storage	59/255 (23.1%)	94 (31.6%)	53 (28.2%)	64 (28.3%)
Water				
Main source of household drinking water improved	155/254 (61.0%)	187 (63.0%)	120 (62.8%)	138 (60.8%)
Treated drinking water to make safer	33/251 (13.1%)	34 (11.6%)	22 (11.6%)	28 (12.4%)
Median one-way walk time to fetch water [IQR], min	10[5,15]	5.5[3,15]	10[5,20]	10[5,15]
Mean water volume collected per person in past 24 h (SD), L	7.8(6.6)	8.6(7.3)	7.7(7.0)	8.5(10.7)
Hygiene				
Handwashing station at household	5/242 (2.1%)	6 (2.1%)	39 (20.2%)	56 (24.8%)
Handwashing station with water and rubbing agent	5/242 (2.1%)	3 (1.0%)	10 (5.2%)	9 (4.0%)
Improved floor*	129 (46.9%)	129 (41.2%)	83 (40.9%)	104 (43.2%)
Median number of chickens [IQR]	6 [1,10]	7 [3,12]	5.5 [2,10]	6 [1,10]
Livestock observed inside home	104 (40.5%)	117 (38.9%)	85 (43.1%)	96 (40.9%)
Feces observed in the yard	84 (32.7%)	118 (39.2%)	72 (36.4%)	76 (32.3%)
Diet quality and food security				
Household meets minimum Diet Diversity Score[58]	106/235 (45.1%)	133/266 (50.0%)	83/178 (46.6%)	94/208 (45.2%)
Median Coping Strategies Index score [IQR][59]	0 [0,6]	0 [0,4]	0 [0,7]	0 [0,4]
Maternal characteristics				
Mean age (SD), years	26.0 (6.5)	26.6 (6.7)	27.0 (6.9)	27.1 (6.6)
Mean height (SD), cm	159.6 (8.3)	160.5 (6.1)	159.3 (9.1)	159.6 (8.4)
Mean mid-upper-arm circumference (SD), cm	26.2 (3.0)	26.7 (3.2)	26.9 (3.5)	26.8 (3.4)
Positive microscopy for *Schistosoma haematobium*	15/252 (6.0%)	31/290 (10.7%)	25/191 (13.1%)	17 (7.4%)
Mean years of schooling completed (SD)	9.4 (1.8)	9.7 (1.7)	9.4 (1.8)	9.5 (1.9)
Median parity [IQR]	2 [1,3]	2 [1,3]	2 [1,3]	2 [1,3]
Married	252 (94.0%)	285 (95.0%)	182 (92.9%)	225 (95.7%)
Employed	16 (6.2%)	33 (11.0%)	32 (16.2%)	21 (9.0%)
Religion				
Apostolic	139 (51.7%)	133 (43.9%)	99 (50.3%)	113 (47.9%)
Other Christian	119 (44.2%)	150 (49.5%)	84 (42.6%)	100 (42.4%)
Other	11 (4.1%)	20 (6.6%)	14 (7.1%)	23 (9.7%)
Infant characteristics				
Female sex	125 (44.8%)	147 (46.5%)	112 (54.4%)	129 (52.7%)
Mean birthweight (SD), kg	3.09 (0.47)	3.13 (0.45)	3.14 (0.46)	3.11 (0.47)
Birthweight <2500 g	26 (9.7%)	15 (5.0%)	13 (6.7%)	25 (10.5%)
Institutional delivery	245 (89.7%)	271 (88.6%)	179 (92.3%)	216 (91.5%)
Vaginal delivery	263 (96.0%)	282 (91.6%)	187 (92.6%)	224 (93.3%)

Baseline variables are shown for 1046 infants and their mothers enrolled in the EED substudy who provided at least one specimen of stool or blood at the 18-month endline visit. Maternal and household data were collected about 2 weeks after consent was recorded (approximately 14 gestational weeks). Baseline for infants was at birth. Data are n or n (%), unless otherwise specified. Where n not stated, <7% of data missing.

SHINE, Sanitation, Hygiene, Infant Nutrition Efficacy trial; EED, environmental enteric dysfunction; SOC, standard of care; IYCF, infant and young child feeding; WASH, water, sanitation, and hygiene. IQR, interquartile range; min, minutes; SD, standard deviation; L, litres; cm, centimetres; kg, kilograms; g, grams.

*Improved floor defined as concrete, brick, cement, or tile; unimproved floor defined as mud, earth, sand, or dung.

### Intervention uptake

At the 12-month study visit, among EED households in the WASH arms, 97% received ventilated improved pit latrines, almost all received handwashing stations and play mats, more than 96% received play yards, and >80% received 80% or more of the planned deliveries of soap and chlorine solution (Table 3). Among households in the IYCF arms, approximately 80% received ≥80% of planned deliveries of the SQ-LNS. Across all intervention groups ≥92% completed the intervention modules. A median of 15 out of 15 scheduled intervention visits between enrolment and 12 months were received by mothers in all groups, with the exception of the WASH-only arm (median[IQR] 14[14–15].

**Table 3:** Intervention delivery and participant uptake in the SHINE EED substudy by treatment group.

	SOC	IYCF	WASH	IYCF plus WASH	Non-WASH^a^	Combined WASH^a^	Non-IYCF^a^	Combined IYCF^a^
**Fidelity of intervention delivery**								
Children with 18-month outcomes (on whom inferences are based), n	279	316	206	245	595	451	485	561
WASH Supplies								
SHINE-installed ventilated improved pit latrine	NA	NA	203 (98.5%)	238 (97.1%)	NA	441 (97.8%)	NA	NA
Two handwashing stations (Tippy Taps) delivered	NA	NA	205 (99.5%)	245 (100%)	NA	450 (99.8%)	NA	NA
Baby mat Delivered	NA	NA	200 (97.1%)	244 (99.6%)	NA	444 (98.4%)	NA	NA
Play yard Delivered	NA	NA	199 (96.6%)	237 (96.7%)	NA	436 (96.7%)	NA	NA
Median liquid soap deliveries [IQR]^b^	NA	NA	20 [19–20]	20 [20–20]	NA	20 [19–20]	NA	NA
Received ≥80% of expected soap deliveries	NA	NA	175 (85.0%)	218 (89.0%)	NA	393 (87.1%)	NA	NA
Median Water Guard deliveries [IQR]^c^	NA	NA	15 [14–15]	15 [15–15]	NA	15 [15–15]	NA	NA
Received ≥80% of expected Water Guard deliveries	NA	NA	175 (85.0%)	216 (88.2%)	NA	391 (86.7%)	NA	NA
IYCF Supplies								
Median deliveries of small-quantity lipid-based nutrient supplement [IQR]^d^	NA	13 [12–13]	NA	13 [13–13]	NA	NA	NA	13 [13–13]
Received ≥11 (80% of expected) deliveries of small-quantity lipid-based nutrient supplement	NA	254 (80.4%)	NA	209 (85.3%)	NA	NA	NA	463 (82.5%)
Behavior change modules								
Median intervention modules [IQR]^c^	15 [14–15]	15 [14–15]	14 [14–15]	15 [15–15]	15 [14–15]	15 [14–15]	15 [14–15]	15 [15–15]
Completed intervention modules	5114/5522 (92.6%)	7877/8350 (94.3%)	5203/5632 (92.4%)	7289/7680 (94.9%)	12991/13872 (93.6%)	12492/13312 (93.8%)	10317/11154 (92.5%)	15166/16030 (94.6%)
**Participant uptake of promoted behaviors at the 12-month visit**								
WASH behaviors								
Mothers with outcomes at 12 months and 18 months, n	239	282	176	221	521	397	415	503
Children with outcomes at 12 months and 18 months, n	243	284	179	225	527	404	422	509
Household members who defecate in the open	487/1029 (47.3%)	516/1346 (38.3%)	2/869 (0.2%)	11/1162 (0.9%)	1003/2375 (42.2%)	13/2031 (0.6%)	NA	NA
Any latrine at household	80 (33.2%)	116 (42.0%)	178 (100%)	225 (100%)	196 (37.9%)	403 (100%)	NA	NA
Improved latrine at household	64 (26.6%)	97 (35.1%)	178 (100%)	225 (100%)	161 (31.1%)	403 (100%)	NA	NA
Improved latrine with well-trodden path not shared with other households and not used for storage	53 (22.0%)	75 (27.2%)	154 (86.5%)	200 (88.9%)	128 (24.8%)	354 (87.8%)	NA	NA
Handwashing station at household	19 (8.2%)	26 (9.6%)	177 (98.9%)	220 (97.8%)	45 (8.9%)	397 (98.3%)	NA	NA
Handwashing station with water and rubbing agent at household	9 (3.9%)	7 (2.6%)	150 (86.7%)	187/210 (89.0%)	16 (3.2%)	337 (88.0%)	NA	NA
Ever treats drinking water to make safer	31 (12.9%)	28 (10.1%)	167 (93.8%)	203 (90.2%)	59 (11.4%)	370 (91.8%)	NA	NA
Disposes water from cleaning infant nappies in a latrine	68 (28.6%)	67 (24.7%)	49 (27.8%)	55 (24.9%)	135 (26.5%)	104 (26.2%)	NA	NA
Play space is visibly clean	NA	NA	164 (94.8%)	200 (92.2%)	NA	364 (93.3%)	NA	NA
Child ever observed to eat soil	179 (74.3%)	188 (66.4%)	38 (21.2%)	62 (27.6%)	367 (70.0%)	100 (24.8%)	NA	NA
Child ever observed to eat chicken feces	65 (27.0%)	53 (18.7%)	5 (2.8%)	7 (3.1%)	118 (22.5%)	12 (3.0%)	NA	NA
IYCF Behaviors								
Child still breastfeeding	237 (97.9%)	277 (97.9%)	175 (97.8%)	219 (97.3%)	NA	NA	412 (97.9%)	496 (97.6%)
Mother reports correct ways to feed child during and after illness	168 (69.4%)	220 (78.6%)	123 (69.5%)	170 (75.9%)	NA	NA	NA	NA
Infant diet met minimum dietary diversity in past 24h	NA	NA	NA	NA	NA	NA	291 (69.5%)	390 (77.4%)
Infant consumed iron-rich food in past 24h	129 (53.5%)	186 (67.1%)	91 (52.3%)	158 (71.5%)	NA	NA	220 (53.0%)	344 (69.1%)
Infant consumed animal source food in past 24h	163 (67.4%)	195 (68.9%)	116 (65.5%)	150 (66.7%)	NA	NA	279 (66.6%)	345 (67.9%)
Infant consumed vitamin-A-rich food in past 24h	165 (68.2%)	224 (79.2%)	119 (66.5%)	178 (79.8%)	NA	NA	284 (67.5%)	402 (79.4%)
Nutributter consumed in past 24h	NA	265 (94.3%)	NA	202 (89.8%)	NA	NA	NA	467 (92.3%)

Data are n or n (%), unless otherwise specified. The denominator for indicators of fidelity of intervention delivery are the number of children who provided outcomes and a plasma or stool specimen at 18 months. The denominator for indicators of participant uptake of promoted behaviours at the 12-month visit are the number of women (for household-level indicators) and children (for child-level indicators) who provided 12-month and 18-month outcomes. Where n not stated, <7% of data missing.

Village health workers were scheduled to visit households monthly to deliver 30 sachets of a small-quantity lipid-based nutrient supplement (sufficient to provide 20g per day for the infant), 1 L of liquid soap, and 150mL (one bottle) of Water Guard for families of fewer than five people (two bottles for families of five or more people). The combined WASH group comprised the group receiving only the WASH intervention and the group receiving the WASH + IYCF intervention, whereas the non-WASH group comprised the two arms not including WASH (SOC and IYCF). The combined IYCF group comprised the two IYCF-containing arms (IYCF and IYCF+WASH), whereas the non-IYCF group comprised the two arms not including IYCF (SOC and WASH).

SHINE, Sanitation, Hygiene, Infant Nutrition Efficacy trial; EED, environmental enteric dysfunction; SOC, standard of care; IYCF, infant and young child feeding; WASH, water, sanitation, and hygiene; NA, not applicable; IQR, interquartile range.

^a^Non-WASH is SOC (n = 279) + IYCF (n = 316); Combined WASH is WASH (n = 206) + IYCF plus WASH (n = 245); Non-IYCF is SOC (n = 279) + WASH (n = 206); Combined IYCF is IYCF (n = 316) + IYCF plus WASH (n = 245)

^b^Maximum of 20 deliveries.

^c^Maximum of 15 deliveries.

^d^Maximum of 13 deliveries.

### Biomarker trends

**Fig 2:**
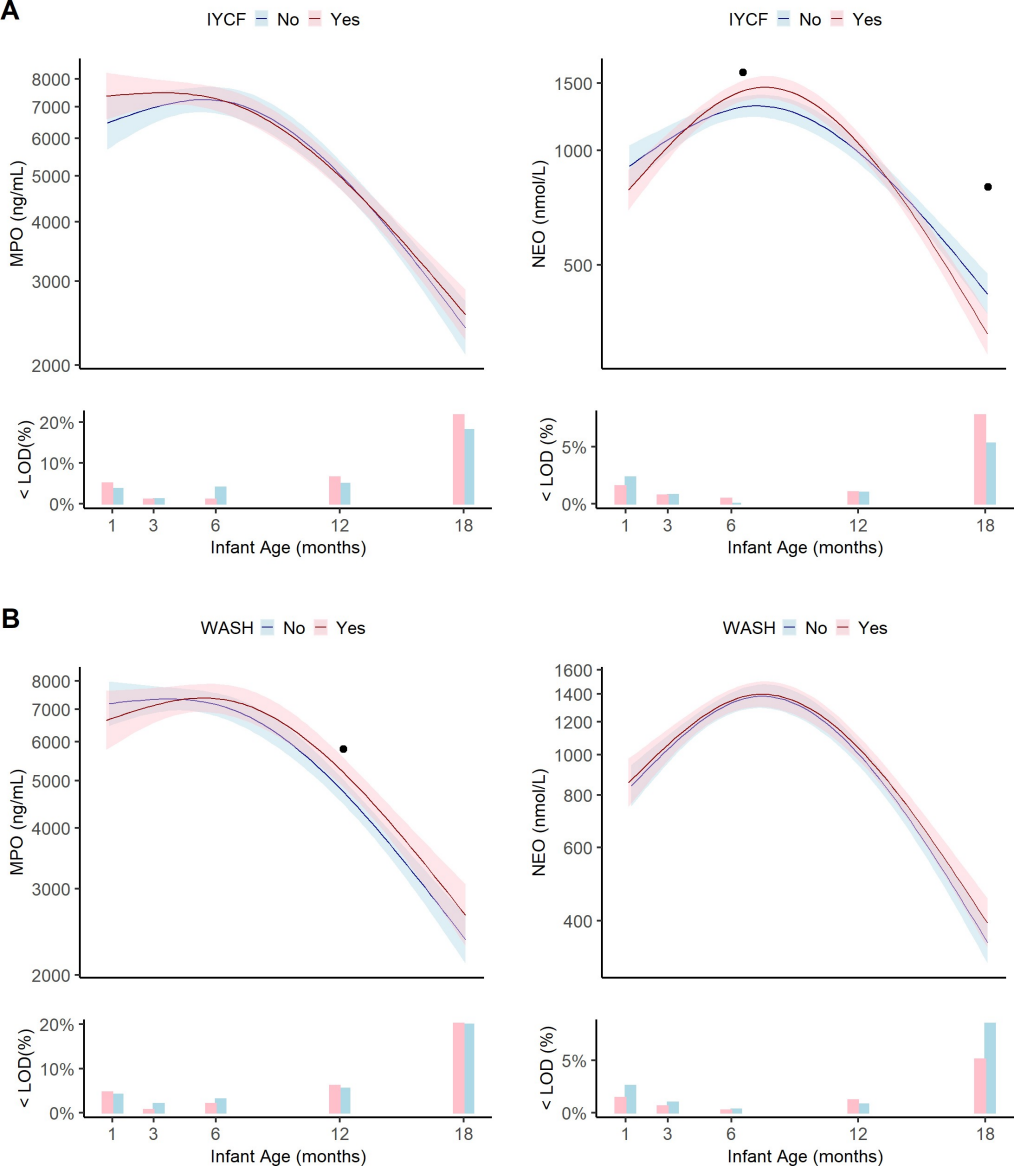
Geometric means, pointwise 95% confidence intervals and the percent of infants below the limit of detection for biomarkers of intestinal inflammation at 1, 3, 6, 12, and 18 months in the EED substudy by combined treatment arm. (A) IYCF vs non-IYCF. (B) WASH vs non-WASH. IYCF, infant and young child feeding; WASH, water, sanitation and hygiene; <LOD(%), percent of samples below the limit of detection. Data were smoothed using generalized additive models with cubic splines and 3 knots. Dot (.) indicates a statistically significant difference between treatment arms at that study visit by Tobit regression. Asterisk (*) indicates statistical significance difference after adjustment for multiple testing. Each visit had a window to enable follow-up if the infant was not seen on the target date. These were: month 1 [4–12 weeks], month 3 [12–25 weeks], month 6 [25–51 weeks], month 12 [51–76 weeks], and month 18 [76–104 weeks]. Median[IQR] infant age in months at each visit was 1.3[1.1,1.9], 3.4[3.2,4.0], 6.4[6.2,7.0], 12.3[12.1,12.5], 18.0[17.8,18.3]. KTR was not measured at 18 months. MPO, myeloperoxidase; NEO, neopterin.

**Fig 3:**
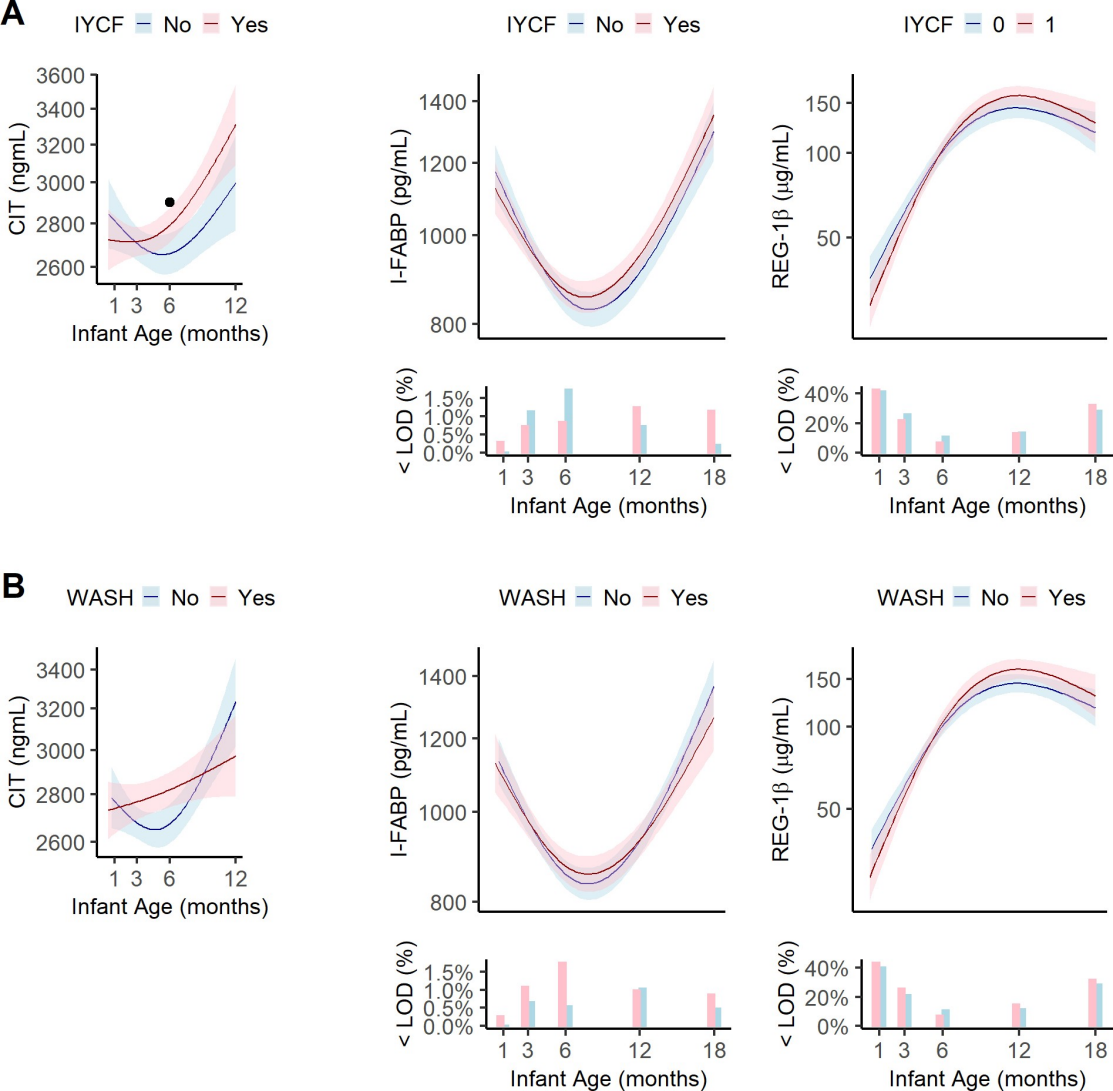
Geometric means, pointwise 95% confidence intervals and the percent of infants below the limit of detection for biomarkers of intestinal damage, biomass and regeneration at 1, 3, 6, 12, and 18 months in the EED substudy by combined treatment arm. (A) IYCF vs non-IYCF. (B) WASH vs non-WASH. IYCF, infant and young child feeding; WASH, water, sanitation and hygiene; <LOD(%), percent of samples below the limit of detection. Data were smoothed using generalized additive models with cubic splines and 3 knots. Dot (.) indicates a statistically significant difference between treatment arms at that study visit by Tobit regression or by GEE estimated linear regression. Asterisk (*) indicates statistical significance after adjustment for multiple testing. Each visit had a window to enable follow-up if the infant was not seen on the target date. These were: month 1 [4–12 weeks], month 3 [12–25 weeks], month 6 [25–51 weeks], month 12 [51–76 weeks], and month 18 [76–104 weeks]. Median[IQR] infant age in months at each visit was 1.3[1.1,1.9], 3.4[3.2,4.0], 6.4[6.2,7.0], 12.3[12.1,12.5], 18.0[17.8,18.3]. Citrulline was not measured at 18 months. CIT, citrulline; I-FABP, intestinal fatty acid binding protein; REG-1β, regenerating gene 1 beta.

**Fig 4:**
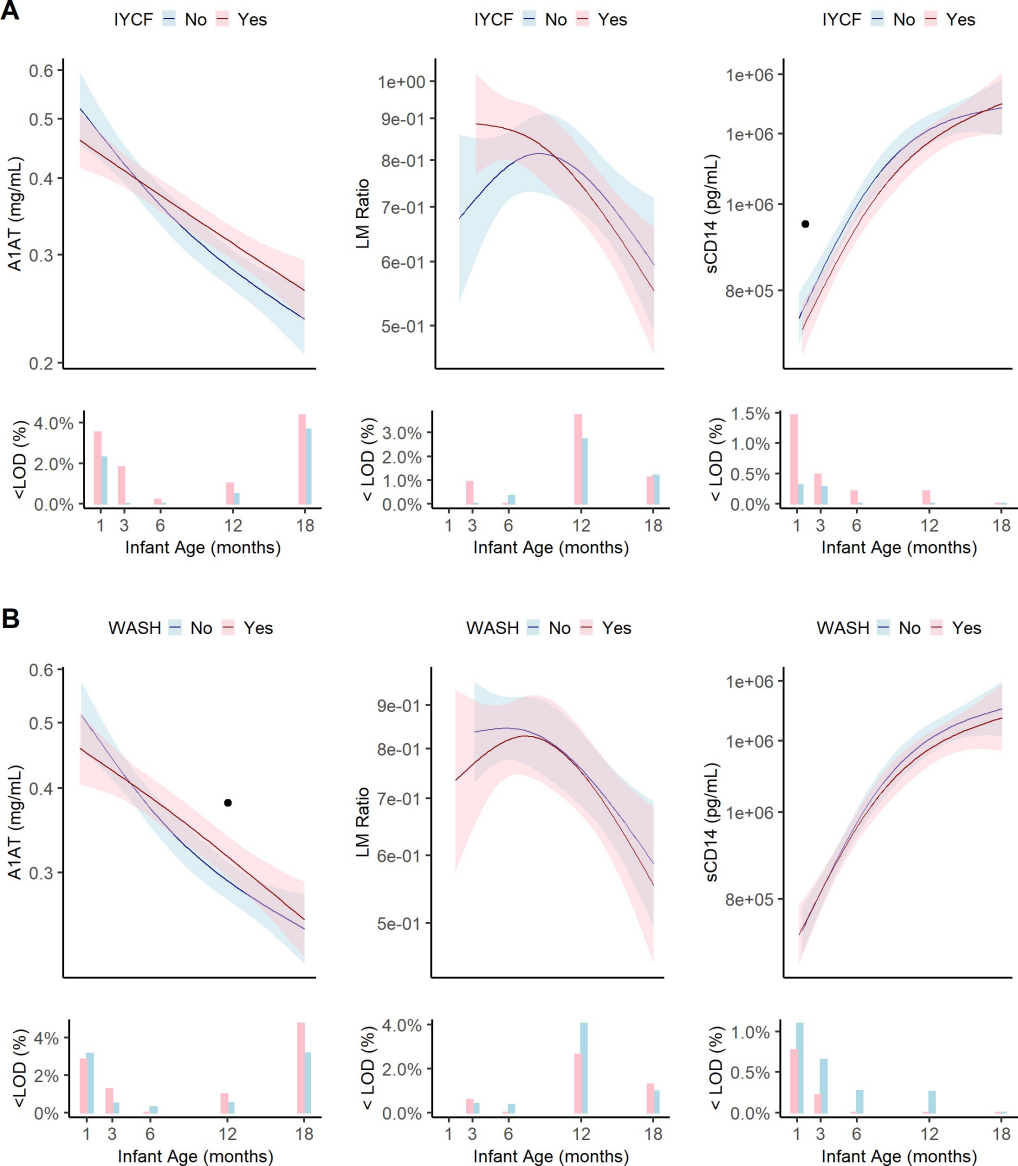
Geometric means, pointwise 95% confidence intervals and the percent of infants below the limit of detection for EED biomarkers of intestinal permeability and microbial translocation at 1, 3, 6, 12, and 18 months in the EED substudy by combined treatment arm. (A) IYCF vs non-IYCF. (B) WASH vs non-WASH. IYCF, infant and young child feeding; WASH, water, sanitation and hygiene; <LOD(%), percent of samples below the limit of detection. Data were smoothed using generalized additive models with cubic splines and 3 knots. Dot (.) indicates a statistically significant difference between treatment arms at that study visit by Tobit regression. Asterisk (*) indicates statistical significance after adjustment for multiple testing. Each visit had a window to enable follow-up if the infant was not seen on the target date. These were: month 1 [4–12 weeks], month 3 [12–25 weeks], month 6 [25–51 weeks], month 12 [51–76 weeks], and month 18 [76–104 weeks]. Median[IQR] infant age in months at each visit was 1.3[1.1,1.9], 3.4[3.2,4.0], 6.4[6.2,7.0], 12.3[12.1,12.5], 18.0[17.8,18.3]. A1AT, alpha-1 antitrypsin; LM, lactulose mannitol; sCD14, soluble CD14.

**Fig 5:**
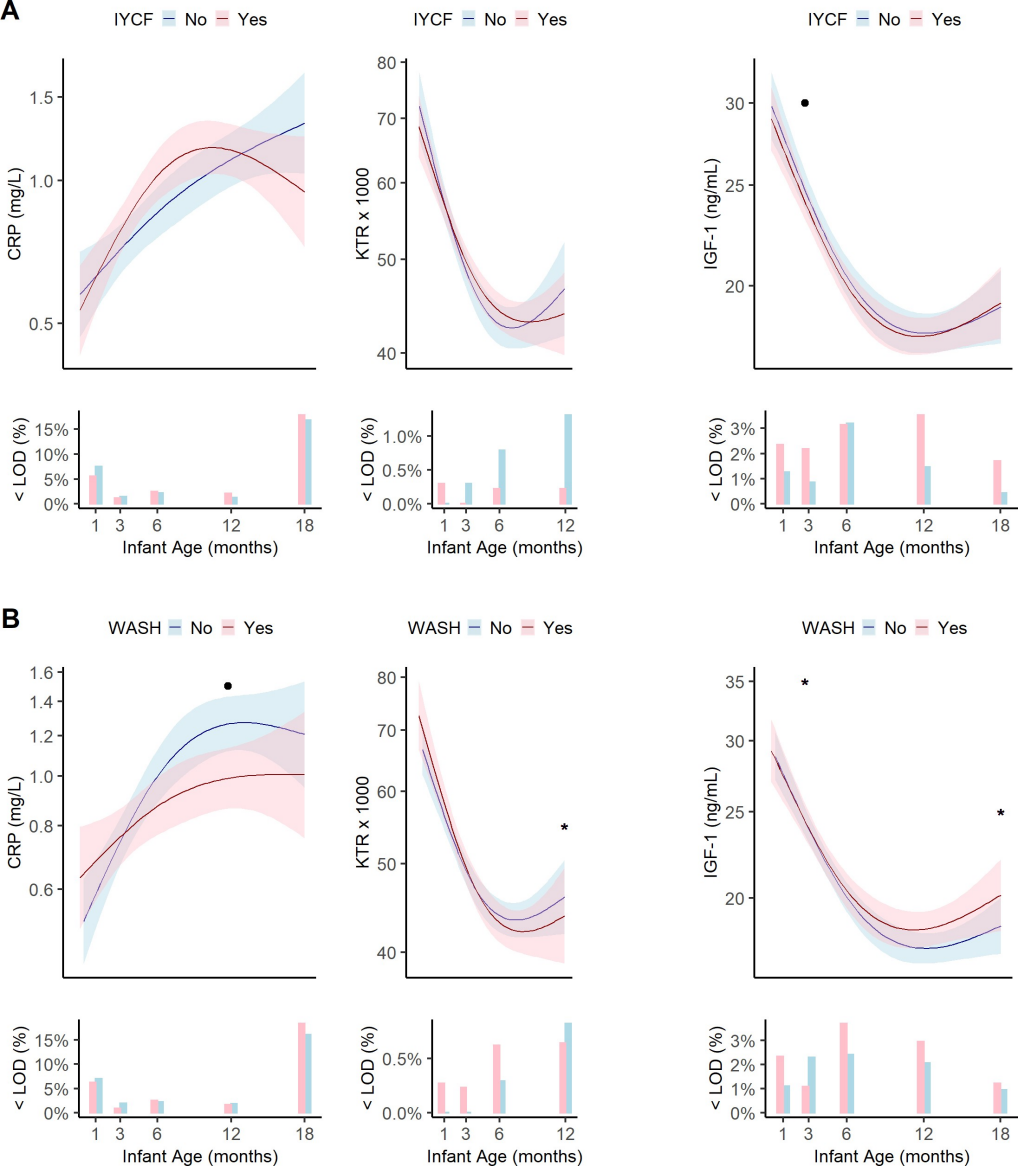
Geometric means, pointwise 95% confidence intervals and the percent of infants below the limit of detection for EED biomarkers of systemic inflammation and growth hormone activity at 1, 3, 6, 12, and 18 months in the EED substudy by combined treatment arm. (A) IYCF vs non-IYCF. (B) WASH vs non-WASH. IYCF, infant and young child feeding; WASH, water, sanitation and hygiene; <LOD(%), percent of samples below the limit of detection. Data were smoothed using generalized additive models with cubic splines and 3 knots. Dot (.) indicates a statistically significant difference between treatment arms at that study visit by Tobit regression. Asterisk (*) indicates statistical significance after adjustment for multiple testing. Each visit had a window to enable follow-up if the infant was not seen on the target date. These were: month 1 [4–12 weeks], month 3 [12–25 weeks], month 6 [25–51 weeks], month 12 [51–76 weeks], and month 18 [76–104 weeks]. Median[IQR] infant age in months at each visit was 1.3[1.1,1.9], 3.4[3.2,4.0], 6.4[6.2,7.0], 12.3[12.1,12.5], 18.0[17.8,18.3]. CRP. C-reactive protein; KTR, kynurenine:tryptophan ratio; IGF-1, insulin-like growth factor 1.

Changes over time in each biomarker between 1–18 months of age, by randomized treatment arm, are shown in Figs 2–5. Stool biomarkers of intestinal inflammation decreased overall during follow-up, but with some variations in the precise pattern (Fig 2).

Myeloperoxidase was quite stable around geometric mean of 7518ng/mL (95%CI:7014–8059) between 1 and 6 months, then rapidly declined thereafter to 2825ng/mL (95%CI:2669–2991) at 18 months. Neopterin showed a minor increase from 1 to 6 months of age (from 980nmol/L (95%CI:908–1057) to 1283nmol/L (95%CI:1230–1337)), followed by a steep decline to 383nmol/L (95%CI:362–407) at 18 months.

Biomarkers of intestinal damage showed more variable patterns (Fig 3).

Stool REG-1β, a marker of epithelial regeneration, increased linearly from 40.6μg/mL (95%CI:36.4–45.1) at 1 month to 154.6μg/mL (95%CI:145.5–164.2) at 12 months, with a small decrease to 132.9μg/mL (95%CI:123.1–143.6) at 18 months. Plasma citrulline, a marker of small intestinal epithelial mass, was approximately 2744ng/ml (95%CI:2658–2834) from 1 to 6 months, but increased to 3150ng/mL (95%CI:3075–3227) by 12 months. Plasma I-FABP, which reflects small intestinal villous damage, showed a U-shaped pattern, declining from 1031pg/mL (95%CI:993–1021) at 1 month to 913pg/mL (95%CI:892–934) at 6 months, followed by an increase to 1198pg/mL (95%CI:1160–1237) at 18 months.

Markers of intestinal permeability decreased during the 18 month follow-up period. Stool A1AT decreased linearly from 0.47mg/mL (95%CI:0.44–0.51) at 1 month to 0.24mg/mL (95%CI:0.22–0.25) at 18 months (Fig 2). Urinary LM ratio peaked at 6 months (0.84, 95%CI:0.71–0.86) then declined steeply thereafter to 0.49 (95%CI:0.43,0.53) at 18 months. By contrast, plasma biomarkers of systemic inflammation showed opposite patterns (Fig 4).

CRP increased between 1 and 18 months, from 0.63mg/L (95%CI:0.55–0.71) to 1.28mg/L (95%CI:1.14–1.43). However, KTR declined from 64.8 (95%CI: 62.2,67.6) at 1 month to approximately 44.9 (95%CI: 43.6,46.3) between 6–12 months.

Soluble CD14, a marker of microbial translocation, showed a pronounced increase from 769,192pg/mL (95%CI: 741,069–798,383) at 1 month to 1,334,175 pg/mL (95%CI: 1,292,950–1,3767,16) at 18 months. Finally, IGF-1, which is produced by the liver in response to growth hormone, declined rapidly from 27.1 ng/mL (26.0–28.2) at 1 month to a fairly stable minimum around 17.9ng/mL at 12 months (17.4–18.5) (Fig 5).

### Intervention effects

There was no consistent evidence of interaction between IYCF and WASH for any biomarker based on our prespecified criteria(S3 Table), so intervention arms were combined into non-IYCF and IYCF, non-WASH and WASH as previously specified. Overall, there was little evidence of intervention effects on biomarkers after adjustment for multiple comparisons. Relative to the non-IYCF group, plasma sCD14 was 10% lower at 1 month in the IYCF group (Tobit β = 0.90, 95%CI:0.81–1.00) (S13 Table); stool neopterin was 1.11 times higher (1.11, 95%CI:0.99–1.25) and 25% lower (0.75, 95%CI:0.58–0.98) at 6 months and 18 months, respectively (S6 Table); plasma citrulline was 8% higher (1.08, 95%CI:1.02–1.14) at 6 months (S11 Table), and plasma IGF-1 was 9% lower at 3 months (0.91, 95%CI:0.83,1.00) (S15 Table). However, these effects were not statistically significant after adjustment for multiple comparisons.

Relative to the non-WASH group, stool A1AT was 1.17 times higher (1.17, 95%CI:1.01–1.35) (S4 Table) and stool myeloperoxidase was 1.16 times higher (1.16, 95%CI:1.02–1.31) at 12 months in the WASH group (S5 Table) resulting in a higher EE score (0.29, 95%CI:0.02–0.57) (S16 Table). At 12 months, plasma KTR was 8% (0.92, 95%CI:0.86,0.98) lower (S7 Table) and plasma CRP was 24% lower (0.76, 95%CI:0.59–0.98) (S14 Table) in the WASH vs non-WASH groups. Plasma IGF-1 was 11% lower in the WASH group (0.89, 95%CI:0.81–0.98) at 3 months and 1.15 times higher (1.15, 95%CI:1.05–1.25) at 18 months, respectively (S15 Table). The decrease in KTR was due to an 8% decrease (0.92,95%CI:0.87–0.97) in kynurenine at 18 months (S8 Table). Only the decrease in IGF-1 at 3 months, the decrease in kynurenine at 12 months, and the increase in plasma IGF-1 at 18 months in the WASH group remained statistically significant after adjustment for multiple testing. Model results were robust to influential observations.

## Discussion

In this substudy of over 1000 HIV-unexposed infants from the SHINE birth cohort in rural Zimbabwe, we investigated the evolution of EED during infancy and evaluated the impact of improved water, sanitation and hygiene, and improved infant feeding on biomarkers of EED. We measured biomarkers that reflect different aspects of intestinal structure and function, microbial translocation, systemic inflammation and growth hormone activity at 1, 3, 6, 12, and 18 months of age, to capture the entire hypothesized pathway from the gut to growth [33]. Overall, we found dynamic changes in all biomarkers over the first 18 months, likely reflecting changes in environmental exposures, intestinal maturation and adaptation during the first thousand days of life. However, we observed no consistent effects of either the IYCF or WASH interventions on biomarkers during the first 18 months after birth, suggesting that our interventions did not impact EED in this cohort.

We report changes over time for the largest number of EED biomarkers in infants to date. Biomarker trends were similar within EED structural and functional domains but varied between domains. In general, markers of intestinal permeability and intestinal inflammation declined over time, whilst markers of microbial translocation and systemic inflammation increased between 1–18 months of age. Markers of intestinal damage (I-FABP) and repair (REG-1β) generally mirrored each other, and citrulline (a marker of intestinal epithelial mass) increased from around 6 months of age, suggesting a dynamic process of epithelial turnover and regeneration in response to enteric insults during infancy. The increase in citrulline at 6 months may also be attributable to the introduction of solid foods around that time, since dietary supplementation can increase plasma citrulline [60]. The higher citrulline concentration in the IYCF group compared to non-IYCF when the SQ-LNS intervention was introduced as 6 months is also consistent with this; however, the higher plasma citrulline in the IYCF group was not significant after correction for multiple comparisons. EED permeability marker A1AT declined linearly through the first 18 postnatal months, while markers of intestinal inflammation (myeloperoxidase and neopterin) remained relatively stable during the first half of infancy and only declined after 6 months of age. The KTR is influenced by tryptophan catabolism through the kynurenine pathway, regulated by the rate-limiting enzyme indoleamine 2,3-dioxygenase 1 (IDO1), which is induced by inflammatory cytokines. Since IDO1 has a wide tissue distribution, including in the digestive tract [61], its activity can be influenced by both systemic and intestinal inflammation [62,63]; furthermore, concentrations of tryptophan may be governed by both dietary intake and microbiome activity [64]. The KTR therefore represents an integrated measure of multiple metabolic and inflammatory processes, and the trend in KTR during infancy differs by geographic setting [37]. In our study, the KTR decreased markedly from 1 to 6 months, similar to previous findings in Peruvian infants [37]. Soluble CD14 and CRP both increased linearly during the 18 months of follow-up. Soluble CD14 is a marker of monocyte activation, predominantly in response to lipopolysaccharide (LPS) stimulation via Toll-like receptor 4. We were unable to measure LPS directly in young infants, in whom blood could not always be taken without contamination from environmental LPS, and we are unable to ascertain the source of LPS that triggers sCD14 elaboration. It seems paradoxical that intestinal permeability declined over time, whilst sCD14 –potentially being produced in response to translocated bacterial products from the gut–increased during the same period. However, lipopolysaccharide may also arise from other sources, such as respiratory exposure to biomass fuels, so sCD14 likely reflects the composite inflammatory milieu arising from multiple sources of bacterial products. Also, A1AT reflects leakage of large proteins from the systemic circulation into the gut and does not reflect the pore size of LPS translocation in the other direction, so these two biomarkers would not be expected to be directly related. Consistent with the rise in sCD14 was a progressive increase in CRP, which reflects low-grade chronic inflammation that develops over time. CRP is produced by the liver in response to stimulation by interleukin-6 and reflects innate immune activation through pattern recognition receptors. Like sCD14, the drivers of CRP remain uncertain and are likely multiple, including bioactive bacterial products from the gut. There is accumulating evidence that low-grade chronic inflammation underlies stunting and other long-term chronic diseases [65]. IGF-1, which is produced by the liver in response to growth hormone stimulation, declined rapidly up to age 12 months, and stabilised thereafter. Overall, IGF-1 concentrations were very low compared to cohorts of European infants [66,67]. Since IGF-1 mediates the effects of growth hormone at the growth plate, these low levels, which decline further over infancy, may be reflective of the population-level linear growth faltering that occurred in this cohort [46]. IGF-1 values declined as CRP increased, consistent with observations from a previous Zimbabwean birth cohort [65], and suggestive of growth hormone resistance due to chronic inflammation, as is seen in clinical disorders such as Crohn’s disease [68,69]. Overall, these biomarker trends show an intense period of adaptation in multiple physiological processes during a key phase of linear growth. The Mal-ED study also reported trends from birth to 24 months of age for A1AT, myeloperoxidase and neopterin, with similar patterns to those observed in our cohort [44]. These values are very similar to the values observed in Zimbabwean infants, and far exceed values in healthy reference populations in high-income countries [70–72].

Overall, we found few effects of the randomized interventions on biomarkers in this large cohort of infants. Compared to the non-IYCF group, we observed a decrease in neopterin at 18 months (β:0.74, 95%CI:0.57–0.96) in the IYCF group after adjusting for multiple testing. Neopterin is a marker of monocyte and macrophage activation by T-helper 1 (TH1) cells in response to infection. An improved nutrient supply can reduce Th1 cell cytokine production to achieve a more balanced inflammatory response [73–75]. Compared to non-WASH, we observed a small decrease in kynurenine at 12 months and an increase in IGF-1 at 18 months in the WASH group after multiple testing correction, but no impact on any other biomarkers. Both effects are small, and the clinical relevance at these ages is unclear.

Overall, the lack of consistent change in any intestinal biomarker suggests that the WASH intervention did not prevent or ameliorate EED, at least as measured by the best currently available biomarkers. This reinforces our previous report that WASH also had very limited effect on enteropathogen carriage [76]. By contrast, WASH Benefits Bangladesh [43] reported that children in all intervention groups had lower intestinal permeability (lactulose and mannitol) and inflammation (neopterin) at age 3 months compared to the control group. Diarrhoea prevalence, *E*.*coli* contamination of water, and *Giardia duodenalis* prevalence were also reduced in this trial [77,78], which is consistent with the hypothesis that EED is caused by environmental exposure to enteric pathogens. However, by age 28 months markers of intestinal permeability (A1AT and lactulose excretion) and inflammation (myeloperoxidase) were increased in the WASH and nutrition arms, making the impact of the trial interventions difficult to ascertain. A smaller randomized trial of community-wide WASH reported a decrease in myeloperoxidase in children 1–5 years of age after 2 years of follow-up [79]. This study also reported significant improvements in access to piped water. There was no impact on soil-transmitted helminth infections, and other indicators of enteric pathogen exposure were not reported [78]. The impact of nutritional interventions have also been inconsistent [19,22,26,27,30,80].

We have recently asserted that the elementary WASH interventions implemented in SHINE did not reduce faecal exposure sufficiently to improve growth [46,81]. Our analyses of the EED substudy, and our prior analysis of enteropathogens [76], are both entirely consistent with this conclusion. Investment in WASH interventions below a minimum threshold may be insufficient to produce noticeable health gains [82], although there are clearly important benefits of WASH for human dignity, women’s time and safety. Community-level improvements in WASH coverage are associated with reductions in environmental faecal contamination [83]. In addition, exposure to animal rather than human excreta may represent a larger source of faecal contamination [84]. Animal exposure has been associated with EED biomarkers of intestinal permeability and inflammation [85]. Whilst we tried to intervene in SHINE to reduce contact between children and animal faeces through geophagia, by providing a play mat and yard as a safe space for children, our intervention was likely insufficient to prevent microbial exposure through this and other routes. A better understanding of faecal exposure pathways [86–88], places of exposure [89], and patterns of exposure to different enteric pathogens [90] may be important to develop more comprehensive WASH interventions. Collectively, the findings from SHINE support our assertion that “transformative WASH” is needed to yield child health gains [81]. Transformative WASH refers to more effective interventions that radically reduce faecal contamination in the household environment [81,91]. These may include a safe and continuous supply of piped household water; high community-wide sanitation coverage; complete separation of animal faeces from the child’s environment; and alternative behaviour-change modalities, as we have recently discussed in detail [81,91]. Proof-of-concept trials that evaluate the impact of transformative WASH on biomarkers of EED and on linear growth are still needed to test the hypothesis that EED underlies stunting and can be prevented through effective reductions in faecal-oral transmission. Our current findings therefore do not suggest that WASH is unimportant for prevention of EED and promotion of linear growth; rather, they suggest that much more investment in WASH is needed to prevent EED and restore healthy growth.

This is the largest cohort to date in which such an extensive range of EED biomarkers has been measured during a crucial period of growth and development in a sub-Saharan African setting. Embedding this substudy in a randomized trial has allowed us to ascertain the effects of WASH and IYCF on these biomarkers during this period, with laboratory analysts who were masked to intervention arm. Some limitations of our analyses are notable. First, this was a substudy from a larger cluster-randomized controlled trial. Although baseline variables were largely balanced across trial arms for the infants included in this analysis, increasing confidence in the internal validity of our findings, there were more mothers from households with a more diverse diet and slightly better coping strategy index, meaning the external validity of these substudy results is unclear. Second, there was less frequent specimen collection at the earliest study visits (1 and 3 months), due to the practice of mothers moving from their homestead in the perinatal period, which may have limited the power to detect statistically significant differences between intervention groups at the youngest ages. Third, EED is a difficult condition to identify. There is no accepted case definition, and available biomarkers have limitations. For example, methods used to undertake and analyse the lactulose-mannitol test differ between studies, as reviewed extensively elsewhere [18]. Biomarkers are imperfect at capturing intestinal structure and dynamics; for example, I-FABP is predominantly (but not entirely) located small intestinal enterocytes in the villous tips, but has a very short half-life and does not necessarily reflect long-term small intestinal damage. However, we were unable to feasibly or ethically obtain small intestinal biopsies from young infants in rural sub-Saharan Africa. As such, we evaluated intervention effects on individual biomarkers as others have done [43], selecting a wide range of biomarkers that we have previously reviewed in detail [33].

Our analyses illustrate that WASH and nutritional interventions did not prevent EED during infancy in an environment with high levels of faecal contamination [76], where biomarkers of gut damage and inflammation highlight the range of enteric insults that are experienced in low-income countries. The elementary WASH interventions tested in SHINE (pit latrines, handwashing stations not connected to a water source, and point-of-use water chlorination) are insufficient to prevent this pervasive and chronic enteric pathology, and transformative solutions are required.

## Members of SHINE trial team

Jean H. Humphrey, Andrew D. Jones, Amee Manges, Goldberg Mangwadu, John A. Maluccio, Mduduzi N. N. Mbuya, Lawrence H. Moulton, Robert Ntozini, Andrew J. Prendergast, Rebecca J. Stoltzfus, James M. Tielsch, Cynthia Chasokela, Ancikaria Chigumira, William Heylar, Preston Hwena, George Kembo, Florence D. Majo, Batsirai Mutasa, Kuda Mutasa, Philippa Rambanepasi, Virginia Sauramba, Naume V. Tavengwa, Franne Van Der Keilen, Chipo Zambezi, Dzivaidzo Chidhanguro, Dorcas Chigodora, Joseph F. Chipanga, Grace Gerema, Tawanda Magara, Mandava Mandava, Tafadzwa Mavhudzi, Clever Mazhanga, Grace Muzaradope, Marian T. Mwapaura, Simon Phiri, Alice Tengende, Cynthia Banda, Bernard Chasekwa, Leah Chidamba, Theodore Chidawanyika, Elisha Chikwindi, Lovemore K. Chingaona, Courage K. Chiorera, Adlight Dandadzi, Margaret Govha, Hlanai Gumbo, Karen T. Gwanzura, Sarudzai Kasaru, Rachel Makasi, Alois M. Matsika, Diana Maunze, Exevia Mazarura, Eddington Mpofu, Johnson Mushonga, Tafadzwa E. Mushore, Tracey Muzira, Netsai Nembaware, Sibongile Nkiwane, Penias Nyamwino, Sandra D. Rukobo, Thompson Runodamoto, Shepherd Seremwe, Pururudzai Simango, Joice Tome, Blessing Tsenesa, Umali Amadu, Beauty Bangira, Daniel Chiveza, Priscilla Hove, Horaiti A Jombe, Didymus Kujenga, Lenin Madhuyu, Prince Mandina-Makoni, Naume Maramba, Betty Maregere, Ellen Marumani, Elisha Masakadze, Phathisiwe Mazula, Caroline Munyanyi, Grace Musanhu, Raymond C. Mushanawani, Sibongile Mutsando, Felicia Nazare, Moses Nyarambi, Wellington Nzuda, Trylife Sigauke, Monica Solomon, Tendai Tavengwa, Farisai Biri, Misheck Chafanza, Cloud Chaitezvi, Tsundukani Chauke, Collen Chidzomba, Tawanda Dadirai, Clemence Fundira, Athanasios C. Gambiza, Tatenda Godzongere, Maria Kuona, Tariro Mafuratidze, Idah Mapurisa, Tsitsi Mashedze, Nokuthula Moyo, Charles Musariri, Matambudzo Mushambadope, Tawanda R. Mutsonziwa, Augustine Muzondo, Rudo Mwareka, Juleika Nyamupfukudza, Baven Saidi, Tambudzai Sakuhwehwe, Gerald Sikalima, Jenneth Tembe, Tapiwanashe E. Chekera, Owen Chihombe, Muchaneta Chikombingo, Tichaona Chirinda, Admire Chivizhe, Ratidzai Hove, Rudo Kufa, Tatenda F. Machikopa, Wilbert Mandaza, Liberty Mandongwe, Farirai Manhiyo, Emmanuel Manyaga, Peter Mapuranga, Farai S. Matimba, Patience Matonhodze, Sarah Mhuri, Joice Mike, Bekezela Ncube, Walter T. S. Nderecha, Munyaradzi Noah, Charles Nyamadzawo, Jonathan Penda, Asinje Saidi, Sarudzai Shonhayi, Clemence Simon, Monica Tichagwa, Rachael Chamakono, Annie Chauke, Andrew F. Gatsi, Blessing Hwena, Hillary Jawi, Benjamin Kaisa, Sithembile Kamutanho, Tapiwa Kaswa, Paradhi Kayeruza, Juliet Lunga, Nomatter Magogo, Daniel Manyeruke, Patricia Mazani, Fungai Mhuriyengwe, Farisai Mlambo, Stephen Moyo, Tawanda Mpofu, Mishelle Mugava, Yvonne Mukungwa, Fungai Muroyiwa, Eddington Mushonga, Selestino Nyekete, Tendai Rinashe, Kundai Sibanda, Milton Chemhuru, Jeffrey Chikunya, Vimbai F. Chikwavaire, Charity Chikwiriro, Anderson Chimusoro, Jotam Chinyama, Gerald Gwinji, Nokuthula Hoko-Sibanda, Rutendo Kandawasvika, Tendai Madzimure, Brian Maponga, Antonella Mapuranga, Joana Marembo, Luckmore Matsunge, Simbarashe Maunga, Mary Muchekeza, Monica Muti, Marvin Nyamana, Efa Azhuda, Urayai Bhoroma, Ailleen Biriyadi, Elizabeth Chafota, Angelline Chakwizira, Agness Chamhamiwa, Tavengwa Champion, Stella Chazuza, Beauty Chikwira, Chengeto Chingozho, Abigail Chitabwa, Annamary Dhurumba, Albert Furidzirai, Andrew Gandanga, Chipo Gukuta, Beauty Macheche, Bongani Marihwi, Barbara Masike, Eunice Mutangandura, Beatrice Mutodza, Angeline Mutsindikwa, Alice Mwale, Rebecca Ndhlovu, Norah Nduna, Cathrine Nyamandi, Elias Ruvata, Babra Sithole, Rofina Urayai, Bigboy Vengesa, Micheal Zorounye, Memory Bamule, Michael Bande, Kumbirai Chahuruva, Lilian Chidumba, Zvisinei Chigove, Kefas Chiguri, Susan Chikuni, Ruvarashe Chikwanda, Tarisai Chimbi, Micheal Chingozho, Olinia Chinhamo, Regina Chinokuramba, Chiratidzo Chinyoka, Xaviour Chipenzi, Raviro Chipute, Godfrey Chiribhani, Mary Chitsinga, Charles Chiwanga, Anamaria Chiza, Faith Chombe, Memory Denhere, Ephania Dhamba, Miriam Dhamba, Joyas Dube, Florence Dzimbanhete, Godfrey Dzingai, Sikhutele Fusira, Major Gonese, Johnson Gota, Kresencia Gumure, Phinias Gwaidza, Margret Gwangwava, Winnet Gwara, Melania Gwauya, Maidei Gwiba, Joyce Hamauswa, Sarah Hlasera, Eustina Hlukani, Joseph Hotera, Lovemore Jakwa, Gilbert Jangara, Micheal Janyure, Christopher Jari, Duvai Juru, Tabeth Kapuma, Paschalina Konzai, Moly Mabhodha, Susan Maburutse, Chipo Macheka, Tawanda Machigaya, Florence Machingauta, Eucaria Machokoto, Evelyn Madhumba, Learnard Madziise, Clipps Madziva, Mavis Madzivire, Mistake Mafukise, Marceline Maganga, Senzeni Maganga, Emmanuel Mageja, Miriam Mahanya, Evelyn Mahaso, Sanelisiwe Mahleka, Pauline Makanhiwa, Mavis Makarudze, Constant Makeche, Nickson Makopa, Ranganai Makumbe, Mascline Mandire, Eunice Mandiyanike, Eunice Mangena, Farai Mangiro, Alice Mangwadu, Tambudzai Mangwengwe, Juliet Manhidza, Farai Manhovo, Irene Manono, Shylet Mapako, Evangelista Mapfumo, Timothy Mapfumo, Jane Mapuka, Douglas Masama, Getrude Masenge, Margreth Mashasha, Veronica Mashivire, Moses Matunhu, Pazvichaenda Mavhoro, Godfrey Mawuka, Ireen Mazango, Netsai Mazhata, David Mazuva, Mary Mazuva, Filomina Mbinda, John Mborera, Upenyu Mfiri, Florence Mhandu, Chrispen Mhike, Tambudzai Mhike, Artwell Mhuka, Judith Midzi, Siqondeni Moyo, Michael Mpundu, Nicholas Msekiwa Msindo, Dominic Msindo, Choice Mtisi, Gladys Muchemwa, Nyadziso Mujere, Ellison Mukaro, Kilvera Muketiwa, Silvia Mungoi, Esline Munzava, Rosewita Muoki, Harugumi Mupura, Evelyn Murerwa, Clarieta Murisi, Letwin Muroyiwa, Musara Muruvi, Nelson Musemwa, Christina Mushure, Judith Mutero, Philipa Mutero, Patrick Mutumbu, Cleopatra Mutya, Lucia Muzanango, Martin Muzembi, Dorcus Muzungunye, Valeliah Mwazha, Thembeni Ncube, Takunda Ndava, Nomvuyo Ndlovu, Pauline Nehowa, Dorothy Ngara, Leonard Nguruve, Petronella Nhigo, Samukeliso Nkiwane, Luckson Nyanyai, Judith Nzombe, Evelyn Office, Beatrice Paul, Shambadzirai Pavari, Sylvia Ranganai, Stella Ratisai, Martha Rugara, Peter Rusere, Joyce Sakala, Prosper Sango, Sibancengani Shava, Margaret Shekede, Cornellious Shizha, Tedla Sibanda, Neria Tapambwa, John Tembo, Netsai Tinago, Violet Tinago, Theresa Toindepi, John Tovigepi, Modesta Tuhwe, Kundai Tumbo, Tinashe Zaranyika, Tongai Zaru, Kamurayi Zimidzi, Matilda Zindo, Maria Zindonda, Nyaradzai Zinhumwe, Loveness Zishiri, Emerly Ziyambi, James Zvinowanda, Ekenia Bepete, Christine Chiwira, Naume Chuma, Abiegirl Fari, Samson Gavi, Violet Gunha, Fadzai Hakunandava, Constance Huku, Given Hungwe, Grace Maduke, Elliot Manyewe, Tecla Mapfumo, Innocent Marufu, Chenesai Mashiri, Shellie Mazenge, Euphrasia Mbinda, Abigail Mhuri, Charity Muguti, Lucy Munemo, Loveness Musindo, Laina Ngada, Dambudzo Nyembe, Rachel Taruvinga, Emma Tobaiwa, Selina Banda, Jesca Chaipa, Patricia Chakaza, Macdonald Chandigere, Annie Changunduma, Chenesai Chibi, Otilia Chidyagwai, Elika Chidza, Nora Chigatse, Lennard Chikoto, Vongai Chingware, Jaison Chinhamo, Marko Chinhoro, Answer Chiripamberi, Esther Chitavati, Rita Chitiga, Nancy Chivanga, Tracy Chivese, Flora Chizema, Sinikiwe Dera, Annacolleta Dhliwayo, Pauline Dhononga, Ennia Dimingo, Memory Dziyani, Tecla Fambi, Lylian Gambagamba, Sikangela Gandiyari, Charity Gomo, Sarah Gore, Jullin Gundani, Rosemary Gundani, Lazarus Gwarima, Cathrine Gwaringa, Samuel Gwenya, Rebecca Hamilton, Agnes Hlabano, Ennie Hofisi, Florence Hofisi, Stanley Hungwe, Sharai Hwacha, Aquiiline Hwara, Ruth Jogwe, Atanus Kanikani, Lydia Kuchicha, Mitshel Kutsira, Kumbulani Kuziyamisa, Mercy Kuziyamisa, Benjamin Kwangware, Portia Lozani, Joseph Mabuto, Vimbai Mabuto, Loveness Mabvurwa, Rebecca Machacha, Cresenzia Machaya, Roswitha Madembo, Susan Madya, Sheneterai Madzingira, Lloyd Mafa, Fungai Mafuta, Jane Mafuta, Alfred Mahara, Sarudzai Mahonye, Admire Maisva, Admire Makara, Margreth Makover, Ennie Mambongo, Murenga Mambure, Edith Mandizvidza, Gladys Mangena, Elliot Manjengwa, Julius Manomano, Maria Mapfumo, Alice Mapfurire, Letwin Maphosa, Jester Mapundo, Dorcas Mare, Farai Marecha, Selina Marecha, Christine Mashiri, Medina Masiya, Thembinkosi Masuku, Priviledge Masvimbo, Saliwe Matambo, Getrude Matarise, Loveness Matinanga, John Matizanadzo, Margret Maunganidze, Belinda Mawere, Chipiwa Mawire, Yulliana Mazvanya, Maudy Mbasera, Magret Mbono, Cynthia Mhakayakora, Nompumelelo Mhlanga, Bester Mhosva, Nomuhle Moyo, Over Moyo, Robert Moyo, Charity Mpakami, Rudo Mpedzisi, Elizabeth Mpofu, Estery Mpofu, Mavis Mtetwa, Juliet Muchakachi, Tsitsi Mudadada, Kudakwashe Mudzingwa, Mejury Mugwira, Tarsisio Mukarati, Anna Munana, Juliet Munazo, Otilia Munyeki, Patience Mupfeka, Gashirai Murangandi, Maria Muranganwa, Josphine Murenjekwa, Nothando Muringo, Tichafara Mushaninga, Florence Mutaja, Dorah Mutanha, Peregia Mutemeri, Beauty Mutero, Edina Muteya, Sophia Muvembi, Tandiwe Muzenda, Agnes Mwenjota, Sithembisiwe Ncube, Tendai Ndabambi, Nomsa Ndava, Elija Ndlovu, Eveln Nene, Enniah Ngazimbi, Atalia Ngwalati, Tafirenyika Nyama, Agnes Nzembe, Eunica Pabwaungana, Sekai Phiri, Ruwiza Pukuta, Melody Rambanapasi, Tambudzai Rera, Violet Samanga, Sinanzeni Shirichena, Chipiwa Shoko, More Shonhe, Cathrine Shuro, Juliah Sibanda, Edna Sibangani, Nikisi Sibangani, Norman Sibindi, Mercy Sitotombe, Pearson Siwawa, Magret Tagwirei, Pretty Taruvinga, Antony Tavagwisa, Esther Tete, Yeukai Tete, Elliot Thandiwe, Amonilla Tibugari, Stella Timothy, Rumbidzai Tongogara, Lancy Tshuma, Mirirayi Tsikira, Constance Tumba, Rumbidzayi Watinaye, Ethel Zhiradzango, Esther Zimunya, Leanmary Zinengwa, Magret Ziupfu, Job Ziyambe, James A. Church, Amy Desai, Dadirai Fundira, Ethan Gough, Rukundo A. Kambarami, Cynthia R. Matare, Thokozile R. Malaba, Tatenda Mupfudze, Francis Ngure, Laura E. Smith, Val Curtis, Katherine L. Dickin, Jean-Pierre Habicht, Collen Masimirembwa, Peter Morgan, Gretel H. Pelto, Corinne Sheffner-Rogers, Roslyn Thelingwani, Paul Turner, Lindiwe Zungu, Tariro Makadzange, Hilda A. Mujuru.

## Supporting information

S1 Table Baseline characteristics of mothers and their infants enrolled in the EED sub-study versus those who were not. (XLSX)

S2 Table Baseline characteristics of HIV unexposed infants who provided at least one biological specimen (feces or plasma) at 18 months versus those who did not, in the SHINE EED sub-study. (XLSX)

S3 Table IYCF, WASH and IYCF-by-WASH Interaction Effects by Biomarker and Visit among HIV unexposed infants. (XLSX)

S4 Table Fecal alpha-1 antitrypsin (mg/mL) concentration by SHINE intervention arm and follow-up visit among HIV unexposed infants. (XLSX)

S5 Table Fecal myeloperoxidase (ng/mL) concentration by Combined IYCF and WASH arms and follow-up visit among HIV unexposed infants. (XLSX)

S6 Table Fecal neopterin (nmol/L) concentration by Combined IYCF and WASH arms and follow-up visit among HIV unexposed infants. (XLSX)

S7 Table Plasma kynurenine:tryptophan ratio by Combined IYCF and WASH arms and follow-up visit among HIV unexposed infants. (XLSX)

S8 Table Plasma kynurenine (ng/mL) by Combined IYCF and WASH arms and follow-up visit among HIV unexposed infants. (XLSX)

S9 Table Plasma tryptophan (ng/mL) by Combined IYCF and WASH arms and follow-up visit among HIV unexposed infants. (XLSX)

S10 Table Plasma intestinal fatty acid binding protein (pg/mL) concentration by Combined IYCF and WASH arms and follow-up visit among HIV unexposed infants. (XLSX)

S11 Table Plasma citrulline (ng/mL) concentration by Combined IYCF and WASH arms and follow-up visit among HIV unexposed infants. (XLSX)

S12 Table Fecal regenerating gene 1β (μg/mL) concentration by Combined IYCF and WASH arms and follow-up visit among HIV unexposed infants. (XLSX)

S13 Table Plasma soluble CD14 (pg/mL) concentration by Combined IYCF and WASH arms and follow-up visit among HIV unexposed infants. (XLSX)

S14 Table Plasma C-reative protein (mg/L) concentration by Combined IYCF and WASH arms and follow-up visit among HIV unexposed infants. (XLSX)

S15 Table Plasma insulin-like growth factor 1 (ng/mL) concentration by Combined IYCF and WASH arms and follow-up visit among HIV unexposed infants. (XLSX)

S16 Table Kosek EE score by Combined IYCF and WASH arms and follow-up visit among HIV unexposed infants. (XLSX)

S17 Table Lactulose:Mannitol Ratio by Combined IYCF and WASH arms and follow-up visit among HIV unexposed infants. (XLSX)
